# Ureteral Obstruction by the Vas Deferens after Urostomy

**DOI:** 10.1100/tsw.2010.161

**Published:** 2010-09-01

**Authors:** Subramanian Vaidyanathan, Peter L. Hughes, Bakul M. Soni, Gurpreet Singh

**Affiliations:** ^1^ Regional Spinal Injuries Centre, District General Hospital, Southport, Merseyside, UK; ^2^ Department of Radiology, District General Hospital, Southport, Merseyside, UK; ^3^ Department of Urology, District General Hospital, Southport, Merseyside, UK

**Keywords:** spina bifida, ureters, urostomy, hydronephrosis, vas deferens, paraplegia

## Abstract

A male patient with spina bifida and paraplegia, born in 1968, underwent urostomy in 1973. In 1999, he developed urine infections. Intravenous urography showed bilateral hydronephrosis and hydroureter. This patient continued to get recurrent urine infections. In 2009, computed tomography of the abdomen revealed dilatation of the ureters, but the ureters reverted to normal calibre as they passed forward through the anterior abdominal wall. The vas deferens on either side was crossing and kinking the ureter. Magnetic resonance imaging of the abdomen confirmed that the level of obstruction in both ureters was at the site where the vas deferens crossed the ureter and kinked it. While performing urostomy, the ureters below the crossover by the vas deferens were detached from the bladder and attached to the skin for urinary diversion, thus causing the vas deferens to hook the lower end of the ureters. As the patient gained height and weight, thereby increasing abdominal girth, kinking of the ureters by the vas deferens was accentuated. In hindsight, bilateral midline cutaneous urostomy using the ureters below the crossover by the vas deferens represents a poor surgical technique for urinary diversion.

## CASE PRESENTATION

A British Caucasian male, who was born in 1968 with spina bifida, had undergone urostomy in 1973. The patient had an episode of pyelonephritis (left) in 1989 and in 1999, was getting recurrent urine infections. Intravenous urography, performed in March 2000, showed bilateral calyceal clubbing and dilatation of both ureters. In July 2001, intravenous urography revealed bilateral hydronephrosis and hydroureter; changes were more marked on the left.

This patient attended the spinal unit in May 2009 with a history of feeling unwell. Blood urea was 3.3 mmol/L. Ultrasound of the abdomen, performed on 10 June 2009, showed the right kidney, which appeared normal in size and echogenicity. No calculus disease or hydronephrosis was seen. The left kidney showed mild hydronephrosis. No cause was seen on ultrasound; no evidence of calculus disease in the left kidney. Both kidneys were of normal size.

The patient again attended the spinal unit in June 2009 with a history of feeling unwell. Microbiology of the urine showed growth of coliforms, sensitive to gentamicin, and the patient was prescribed gentamicin for 5 days. He was admitted to the spinal unit again 2 weeks later with a history of severe pain in the left flank. Microbiology of the urine showed coliforms, sensitive to gentamicin. Intravenous urography revealed bilateral excretion of contrast with fullness of the collecting system, pelvis, and ureter bilaterally. Appearances suggested a degree of obstruction at the level of the uretero-conduit anastamosis.

Computed tomography (CT) of the abdomen revealed no opaque renal or ureteric calculus. There was mild dilatation of the left pelvicalyceal system and proximal ureter. As the ureters progressed distally, they became more dilated. The ureters then reverted to normal calibre as they passed anteriorly and superiorly towards the anterior abdominal wall. CT showed the vas deferens on either side crossing and kinking the ureter at the level of obstruction (Figs. 1 and 2).

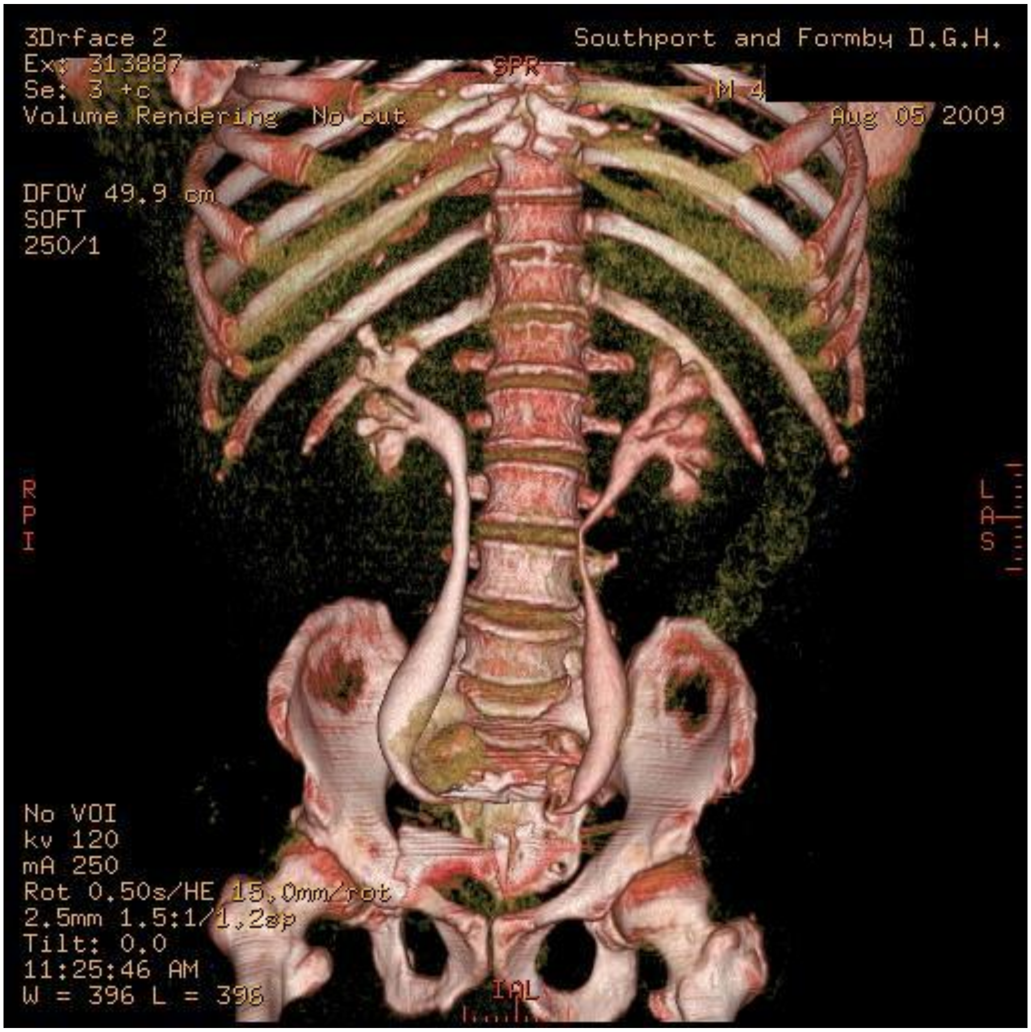

**FIGURE 1**. Surface rendered CT reformatted image of the abdomen in coronal plane, showing the “hooked” course of the lower ureters.

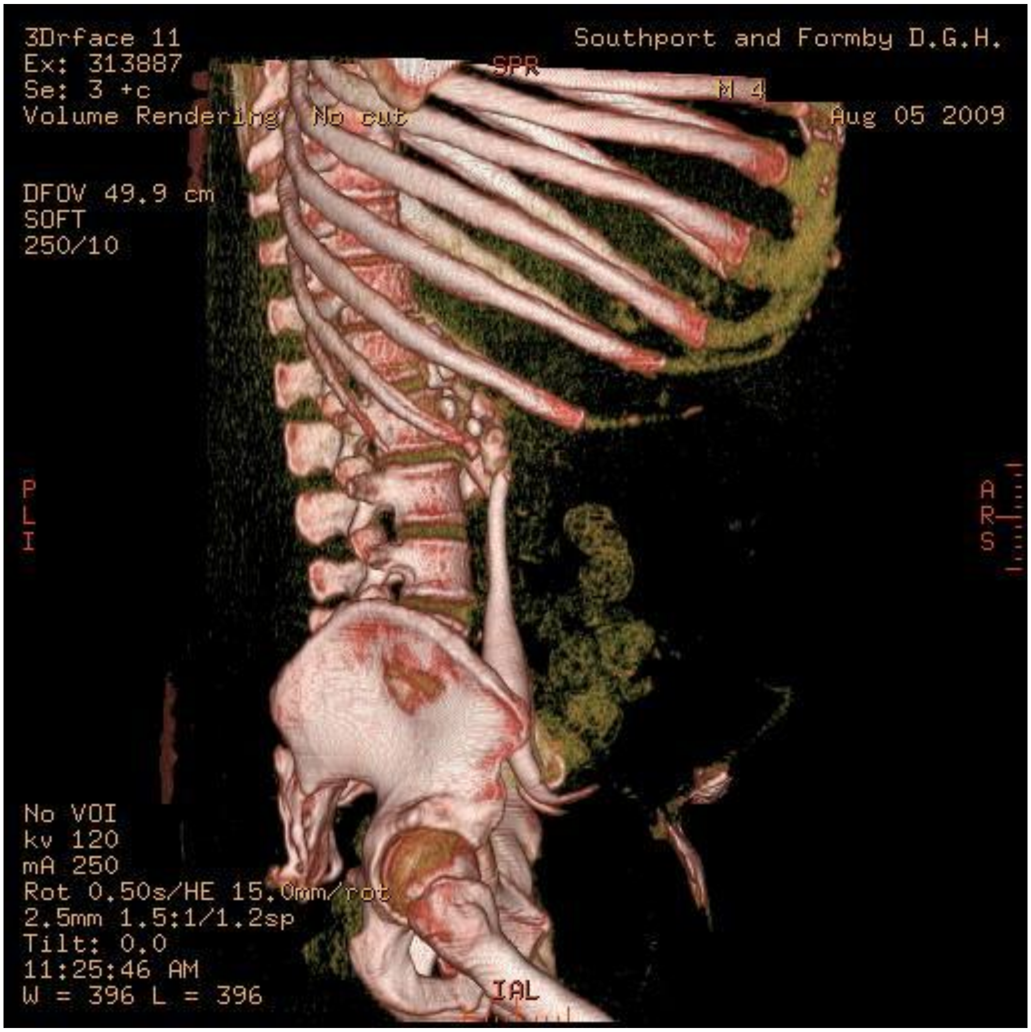

**FIGURE 2**. Surface rendered CT reformatted image of the abdomen in sagittal plane, showing the "hooked" course of the lower ureters.

Magnetic resonance imaging of the abdomen confirmed that the level of obstruction in both ureters was at the site where the vas deferens crossed the ureters, which resulted in kinking bilaterally (Figs. 3–6). There was mild to moderate proximal dilatation of the ureters above this level. Distal to this level, the distal ureters were not dilated and carried on for a short distance superiorly before they entered the abdominal wall at the level of the urostomy.

## COMMENTS: ANATOMY OF VAS DEFERENS

The vas deferens is a constituent of the spermatic cord and traverses the inguinal canal to the abdominal inguinal ring, where it separates from the other structures of the spermatic cord. Then it enters the pelvic cavity, and descends on the medial side of the obliterated umbilical artery and the obturator nerve and vessels. The vas deferens then crosses in front of the ureter and reaches the medial side of ureter, then bends medially and slightly forward between the fundus of the bladder and the upper end of the seminal vesicle (Fig. 7). Reaching the medial side of the seminal vesicle, it is directed downward, gradually approaching the opposite vas. Here it lies between the fundus of the bladder and the rectum.

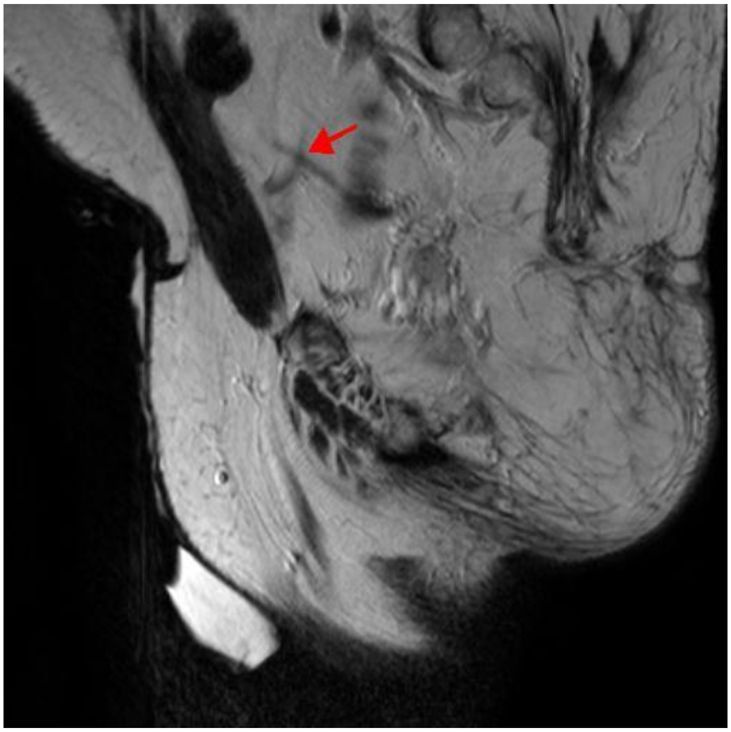

**FIGURE 3**. T-2 weighted sagittal image shows left vas deferens traversing the left ureter (arrow).

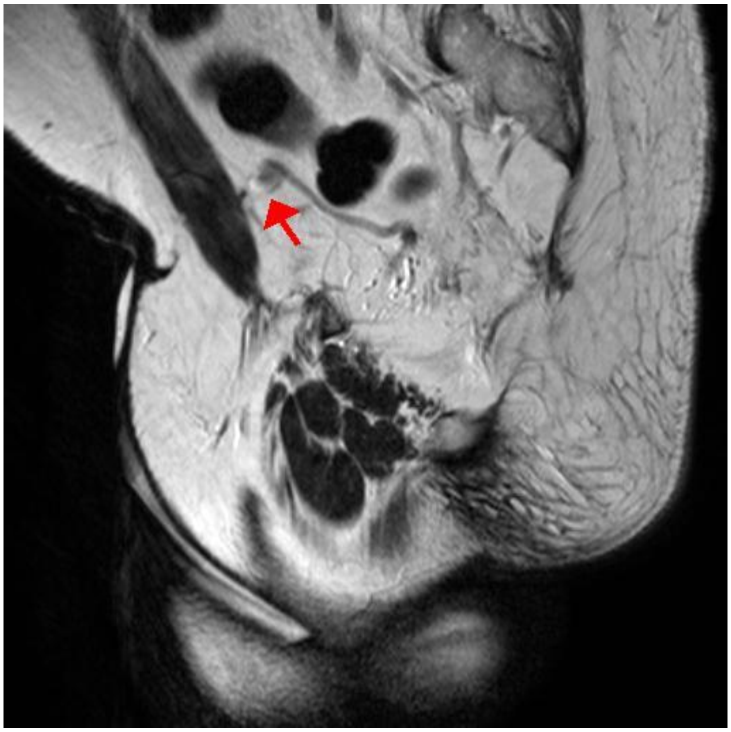

**FIGURE 4.** T-2 weighted sagittal image at the level of right ureter showing the right vas deferens crossing it (arrow).

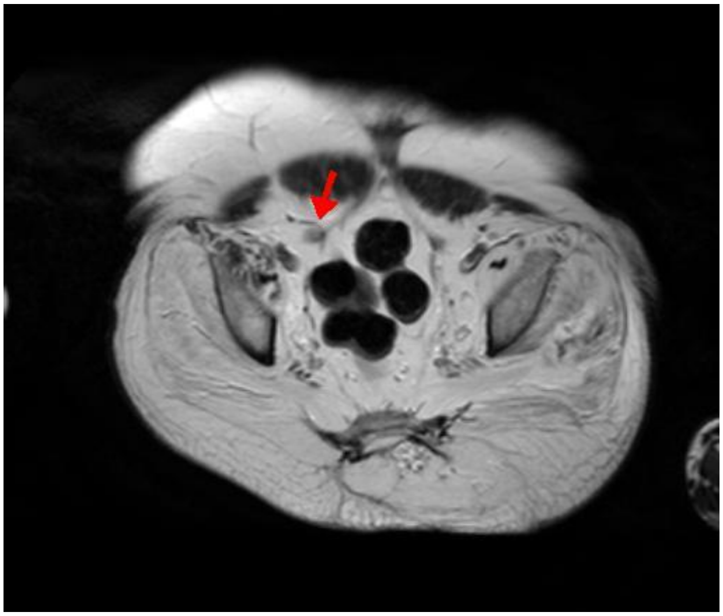

**FIGURE 5.** Axial T-2 weighted image demonstrating the course of the right vas deferens crossing and kinking the lower ureter at the site of incomplete obstruction (arrow).

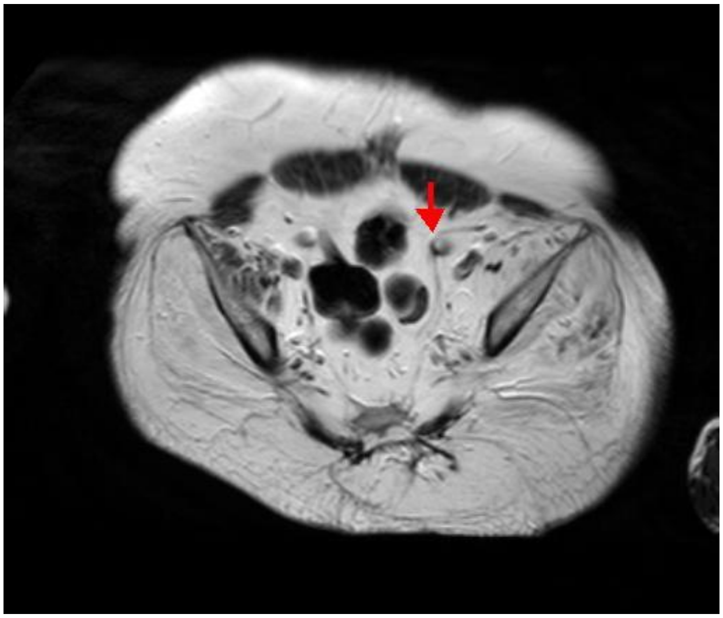

**FIGURE 6.** Axial T-2 weighted image demonstrating the course of the left vas deferens crossing and kinking the lower ureter at the site of incomplete obstruction (arrow).

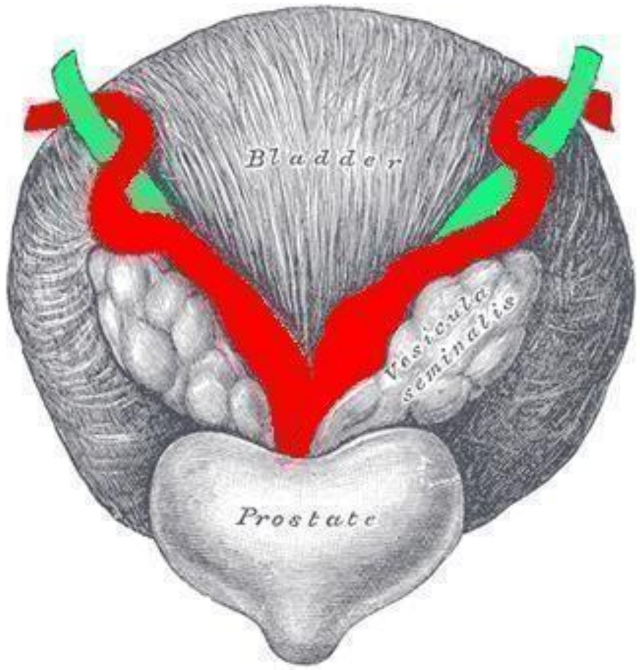

**FIGURE 7.** Diagrammatic representation of the crossover of the ureter (shaded green in this figure) by the vas deferens (shaded red in the above diagram)

In our patient, bilateral midline cutaneous urostomy was performed using the ureters below the crossover by the vas deferens. Following cutaneous urostomy, there was kinking of the ureters by the vas deferens. Ureteral obstruction became symptomatic when this patient grew in height and put on weight, thereby increasing abdominal circumference. This led to accentuation of the kinking of the ureters by the vas deferens. In hindsight, bilateral midline cutaneous urostomy using the ureters below the crossover by the vas deferens represents a poor surgical technique for urinary diversion.

Nguyen and Mitchell[1] reported a rare complication following a difficult unilateral 1-stage Fowler-Stephens orchiopexy resulting in ipsilateral distal ureteral obstruction. The complication arose when the transected spermatic vessels allowed tension to be placed solely on the vas when the testis was placed in the scrotum. The vas in turn compressed the ureter during bladder filling.

Treatment options for kinking of the ureters by the vas deferens include the following:

1. Open or laparoscopic surgery and division of the vas deferens, thus relieving the kinking of the ureters by the vas deferens. Surgery in a paraplegic patient carries a small, but definite risk of complications, such as venous thrombosis and dysmotility of neuropathic bowels. Further, division of the vas deferens will produce azoospermia.
2. Reducing weight and decreasing abdominal circumference, thereby relieving the kinking of the ureters caused by the vas deferens.
3. As a temporary measure, wearing an abdominal corset might help to push the protuberant belly inwards and thereby ease the kinking of the ureters by the vas deferens.

This patient preferred to try nonoperative methods (2 and 3 vide supra), as division of the vas deferens might complicate his chances of begetting another child in the future. During the past 4 months, the patient managed to lose weight and, so far, has not required attendance or admission to the spinal unit for urine infection.

## ACKNOWLEDGMENTS

Supported by an unrestricted grant from Amdipharm Plc.
